# Chikungunya and Dengue Viruses in Travelers

**DOI:** 10.3201/eid1401.070618

**Published:** 2008-01

**Authors:** Loredana Nicoletti, Massimo Ciccozzi, Antonella Marchi, Cristiano Fioretini, Patrizia Martucci, Fortunato D’Ancona, Marta Ciofi degli Atti, Maria Grazia Pompa, Giovanni Rezza, Maria Grazia Ciufolini

**Affiliations:** * Istituto Superiore di Sanità, Rome, Italy; † Ministero della Salute, Rome, Italy

**Keywords:** Chikungunya virus, dengue virus, dengue fever, travelers, letter

**To the Editor:** Chikungunya virus (CHIKV), an arthropod-borne virus transmitted to humans by *Aedes spp*. mosquitoes, was first isolated in Tanzania (Tanganyika) in 1953 (*1*). Various outbreaks have since occurred in Africa, Southeast Asia, and India (*2*).

CHIKV has recently been reported in a large area in the Indian Ocean islands and the Indian subcontinent. After an outbreak in Kenya in 2004, other outbreaks occurred in early 2005 on the Comoros Islands, Réunion, and other islands in the southwestern Indian Ocean; the epidemic then spread to India (*3*,*4*). Molecular analysis showed that the epidemic was caused by a variant of the Central/East African CHIKV genotype (*5*,*6*).

Internet surveillance networks provided information on epidemics in real time, alerting clinicians in the industrialized world to the spread of CHIKV and enabling them to more easily diagnose infection among travelers with fevers (*7*). We report results of diagnostic tests and analysis of predictors of infection among persons in Italy with symptoms suggestive of CHIKV infection who had traveled to potentially affected areas. Dengue virus (DENV) is endemic to many of these areas.

We studied travelers or migrants from areas to which CHIKV infection is endemic (i.e., sub-Saharan Africa) or areas currently affected by outbreaks (i.e., the Indian Ocean islands, India) who had symptoms suggestive of infection (i.e., fever and arthralgia with or without a rash) from January 2006 through March 2007. At least 1 blood sample was collected from each patient and stored at –80°C before testing for CHIKV and DENV. Median lag between onset of symptoms and date of blood collection was 22 days (range 3–179 days). Two samples (acute phase and convalescence phase) were available from 5 patients. Serologic diagnosis of CHIKV infection was determined by hemagglutination inhibition (HI) test and confirmed by plaque-reduction neutralization test (*8*). Serodiagnosis of DENV infection was conducted by using the HI test and an immunoglobulin M ELISA (Focus Diagnostics, Cypress, CA, USA). A case-report form containing information about age, sex, countries visited, travel dates, and date of onset of symptoms was completed for each patient.

Seventy-six persons participated in the study; 55.3% were male, median age was 39 years (range 1–69 years), and most (80.3%) were Italian (Table). A total of 29 (38.2%) were positive for CHIKV, and 13 (17.1%) were positive for DENV; 34 (44.7%) were negative for both viruses. Of the 29 CHIKV-positive persons, 22 (75.9%) had visited the Indian Ocean islands (Mauritius, Réunion, and Madagascar), 5 had visited Asia, and 2 had visited Africa. Travelers from Indian Ocean islands had a higher risk for CHIKV infection than those who had visited Africa (odds ratio [OR] 11.0, 95% confidence interval [CI] 1.60–119.13) or Asia (OR 17.05, 95% CI 4.31–73.05). Persons who had visited Asia had a higher risk for DENV infection (OR 8.36; 95% CI 1.58–81.73) than those who had visited other areas.

**Table:** Characteristics of 76 travelers studied

Characteristic	Chikungunya virus–positive, no. (%)	Dengue virus–positive, no (%)	Seronegative, no. (%)
Total	29 (100.0)	13 (100.0)	34 (100.0)
Sex			
Male	17 (58.6)	6 (46.2)	19 (55.9)
Female	12 (41.4)	7 (53.8)	15 (44.1)
Age, y			
0–35	8 (27.6)	4 (30.8)	16 (47.0)
36–50	11 (37.9)	6 (46.1)	14 (41.2)
>50	10 (34.5)	3 (23.1)	4 (11.8)
Days spent abroad			
0−15	18 (62.1)	4 (30.8)	20 (58.8)
>15	11 (37.9)	9 (69.2)	14 (41.2)
Area visited*			
Africa	2 (6.9)	2 (15.4)	6 (17.6)
African islands	22 (75.9)	0	8 (23.5)
Asia	5 (17.2)	11 (84.6)	20 (58.8)
Nationality			
Italian	23 (79.3)	9 (69.2)	29 (85.3)
Other	6 (20.7)	4 (30.8)	5 (14.7)
Rash			
Yes	19 (65.5)	4 (30.8)	6 (17.6)
No	10 (34.5)	9 (69.2)	28 (82.3)

*Africa, continental Africa; African Islands, Western Indian Ocean islands; Asia, India and Southeast Asia.

The 5 persons who were infected with CHIKV in Asia had visited India (i.e., the most visited country [21 travelers]). However, persons who visited the Indian Ocean islands had a higher risk of being CHIKV positive than those who had visited India (OR 8.8, 95% CI 2.09–39.86). A rash was associated with CHIKV infection and was >8× more likely to be reported by CHIKV-positive persons than CHIKV-negative persons (OR 7.03, 95% CI 2.23–22.93). Moreover, rash was observed in 65% of CHIKV-positive cases and 31% of DENV-positive cases, but the difference was not statistically significant because of the small sample size (OR 4.28, 95% CI 0.88–23.23). None of the other patient’s characteristics was associated with infection with CHIKV or DENV.

A limitation of our study was that only 5 patients had documented seroconversion for CHIKV. However, high titers were found in all but 1 patient (>1,280 in 21 patients and 640 in 2 patients). This patient, who had a titer of 80, was an Italian who had probably not been previously exposed to CHIKV. Thus, the risk for misclassification was low. PCR for early detection of infection was not used because only 3 persons were tested within 10 days of symptom onset. Two of these persons, who were tested 7 days after symptom onset, already had antibodies to CHIKV.

In conclusion, a high proportion of travelers with symptoms of CHIKV infection who returned from areas with outbreaks of this infection or where this virus was endemic were seropositive. A lower proportion of patients had antibodies to DENV. CHIKV-positive patients were more likely to have a rash than those negative for both CHIKV and DENV. As suggested by previous studies (*9*), a rash was more common among CHIKV-positive patients than in DENV-infected patients, but the difference was not significant. Our study suggests that identification of predictors of infection with CHIKV is feasible, although it is complicated by cocirculation of DENV in the same areas.
